# Effects of warming rate, acclimation temperature and ontogeny on the critical thermal maximum of temperate marine fish larvae

**DOI:** 10.1371/journal.pone.0179928

**Published:** 2017-07-27

**Authors:** Marta Moyano, Caroline Candebat, Yannick Ruhbaum, Santiago Álvarez-Fernández, Guy Claireaux, José-Luis Zambonino-Infante, Myron A. Peck

**Affiliations:** 1 Institute of Hydrobiology and Fisheries Science, Center for Earth System Research and Sustainability (CEN), University of Hamburg, Olbersweg 24, Hamburg, Germany; 2 Alfred-Wegener-Institut Helmholtz-Zentrum für Polar- und Meeresforschung, Biologische Anstalt Helgoland, Helgoland, Germany; 3 Université de Bretagne Occidentale, LEMAR (UMR 6539), Unité PFOM-ARN, Centre Ifremer de Bretagne, Plouzané, France; 4 Ifremer, LEMAR (UMR 6539), Unité PFOM-ARN, Centre Ifremer de Bretagne, Plouzané, France; University of Connecticut, UNITED STATES

## Abstract

Most of the thermal tolerance studies on fish have been performed on juveniles and adults, whereas limited information is available for larvae, a stage which may have a particularly narrow range in tolerable temperatures. Moreover, previous studies on thermal limits for marine and freshwater fish larvae (53 studies reviewed here) applied a wide range of methodologies (e.g. the static or dynamic method, different exposure times), making it challenging to compare across taxa. We measured the Critical Thermal Maximum (*CT*_*max*_) of Atlantic herring (*Clupea harengus*) and European seabass (*Dicentrarchus labrax*) larvae using the dynamic method (ramping assay) and assessed the effect of warming rate (0.5 to 9°C h^-1^) and acclimation temperature. The larvae of herring had a lower *CT*_*max*_ (lowest and highest values among 222 individual larvae, 13.1–27.0°C) than seabass (lowest and highest values among 90 individual larvae, 24.2–34.3°C). At faster rates of warming, larval *CT*_*max*_ significantly increased in herring, whereas no effect was observed in seabass. Higher acclimation temperatures led to higher *CT*_*max*_ in herring larvae (2.7 ± 0.9°C increase) with increases more pronounced at lower warming rates. Pre-trials testing the effects of warming rate are recommended. Our results for these two temperate marine fishes suggest using a warming rate of 3–6°C h^-1^: *CT*_max_ is highest in trials of relatively short duration, as has been suggested for larger fish. Additionally, time-dependent thermal tolerance was observed in herring larvae, where a difference of up to 8°C was observed in the upper thermal limit between a 0.5- or 24-h exposure to temperatures >18°C. The present study constitutes a first step towards a standard protocol for measuring thermal tolerance in larval fish.

## Introduction

Performance in fish and other ectotherms is highly controlled by temperature, which sets the pace of physiological processes [1,2]. For that reason, temperature is believed to be largely responsible for the geographical patterns in distribution and abundance of most species [3]. Climate-driven changes, especially global warming, have been correlated to changes in phenology, distribution and abundance of some temperate species [4] and warming may be particularly deleterious for stenothermic animals inhabiting low and high latitudes [5,6]. Despite the importance of understanding thermal physiology to disentangling the mechanisms behind climate-driven changes in populations, basic information on thermal limits is lacking for a large number of marine fish species. Such information is important given the (re-) emphasis of integrating physiological thresholds within models projecting climate impacts [7–9].

**Fig 1:**
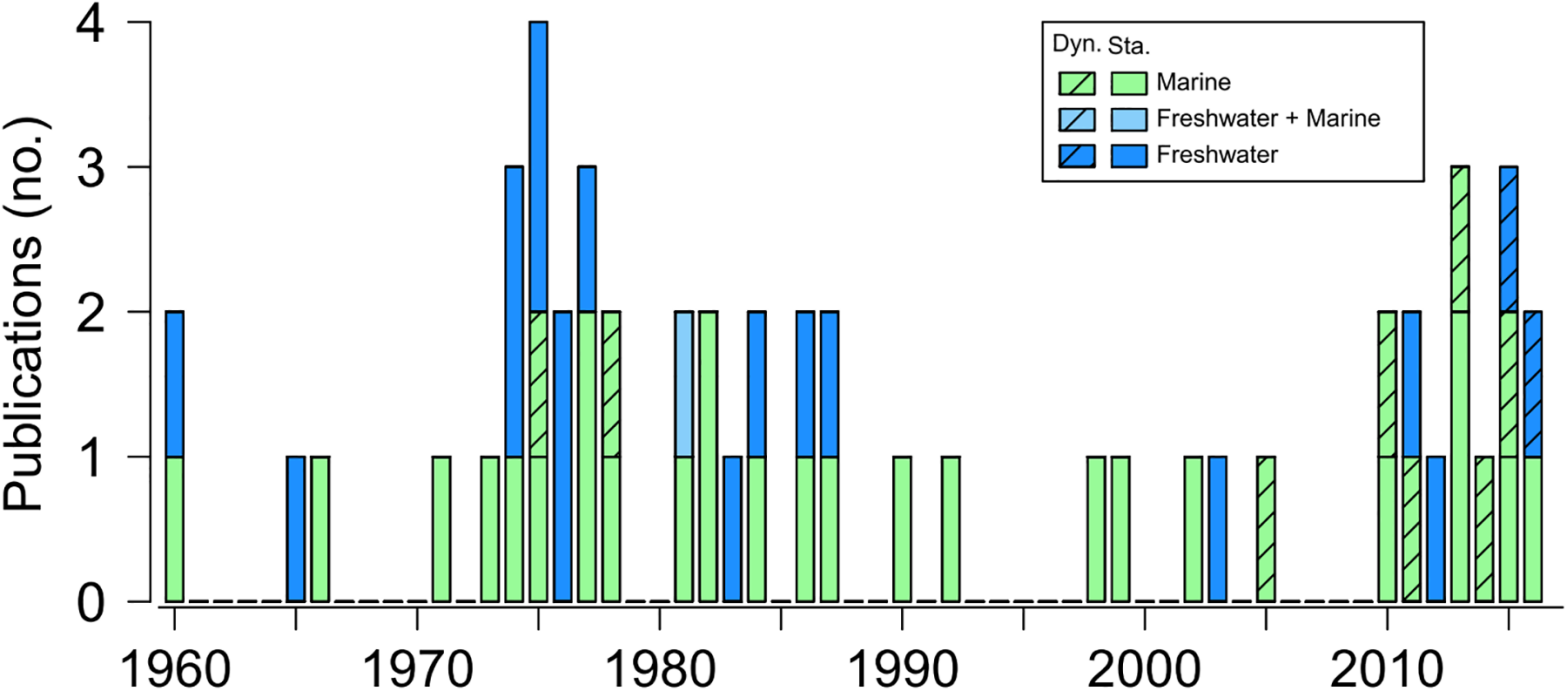
Summary of studies reporting thermal limits for the larvae of freshwater, brackish and marine fish species. Shaded and filled bars are studies using the static (Sta.) and dynamic (Dyn.) method, respectively. See text for further details on both methods.

In fish and other ectotherms, limits to thermal tolerance and the impact of temperature on physiological processes can be stage-specific and larvae are assumed to be a more sensitive life stage (i.e. displaying relatively narrow ranges in tolerable temperatures) compared to juveniles or adults [10]. For instance, the lower latitudinal limit of Arctic cod (*Boreogadus saida*) is unlikely due to adult thermal tolerance and more likely controlled by summer temperatures beyond the tolerable range of larvae [11]. Hence, understanding ontogenetic changes in thermal tolerance is highly relevant in order to identify potential population bottlenecks in future warming scenarios [12]. Unfortunately, relatively few data are available on the thermal tolerance of fish larvae (38 freshwater and 19 marine species, Fig 1, Table 1) compared to juvenile and adult fish (>110 marine species, [9]). Standard protocols are available for juveniles and adults [13,14] but not for larvae which may explain, in part, why far fewer thermal tolerance estimates are available for larvae compared to later life stages.

**Table 1:** Compilation of published studies on thermal limits of marine and freshwater larvae.

Order & Family	Species	Common Name	Larval Habitat	Larval Age / Size	Method					Rearing T (°C)	Thermal limit		Study
					Factor (LT or CT)	Ind. / Groups	Time (h)	Change Rate (°C h^-1^)	End-point		Lower	Upper	
Ord. Acipenseriformes													
Fam. Acipenseridae													
	*Scaphirhynchus albus*	Pallid sturgeon	FW	6–10 mm TL	LT (S)	G	0.1	-	D + LOE	22	-	32.0	[15]
Ord. Atheriniformes													
Fam. Atherinidae													
	*Leurestes sardina*	Gulf grunion	SW	0–30 dph	LT (S)	G	0.5–72.0	-	D	20–30	7.0–8.0	31.0–36.0	[16]
	*Leurestes tenuis*	Californian grunion	SW	0–30 dph	LT (S)	G	0.5–72.0	-	D	20–30	3.0–8.0	32.0–40.0	[16]
Ord. Beloniformes													
Fam. Adrianichthyidae													
	*Oryzias melastigma*	Marine medaka	SW	5 mm	CT (D)	I	168.0	18	LOE	12–32	6.3–12.3	39.9–42.8	[17]
Ord. Clupeiformes													
Fam. Clupeidae													
	*Alosa pseudoharengus*	Alewife	FW	1 dph	LT (S)	G	24.0	abrupt	D	14–15	-	31.0	[18]
	*Brevoortia tyrannus*	Atlantic menhaden	SW	-	LT (S)	G	> 12.0	-	D	7–15	1.5–4.0	-	[19]
	*Clupea harengus*	Atlantic herring	SW	YS	LT (S)	G	24.0	-	D	7, 13		20.5–23.5	[20]
			SW	-	LT (S)	G	0.1–1.0	-	D	8	-	25.0–31.0	[21]
			SW	6–8 mm	LT (S)	G	24.0	-	D	7–15	-2.0–-0.35	22.0–23.5	[22]
Fam. Engraulidae													
	*Engraulis australis*	Australian anchovy	SW	E to YS	LT (S)	G	0.5–24.0	15	D	25–27	-	35.1	[23]
Ord. Cypriniformes													
Fam. Catostomidae													
	*Catostomus commersonii*	White sucker	FW	YS	LT (S)	G	24.0–168.0	-	D	9–21	3.0–6.1	28.0–32.0	[24]
	*Chasmistes brevirostris*	Shortnose sucker	FW	35 dph	LT (S)	G	96.0	-	D	20	-	31.7–32.0	[25]
	*Chasmistes liorus*	June sucker	FW	7 dph	LT + ILT (S)	G	0.1–720.0	abrupt	D	16	-	21.0–33.0	[26]
	*Deltistes luxatus*	Lost River sucker	FW	35 dph	LT (S)	G	96.0	-	D	20	-	31.5–32.0	[25]
Fam. Cyprinidae													
	*Agosia chrysogaster*	Longfin dace	FW	22 dph	CT (D)	I	-	42	LOE	18–30	-	33.5–39.7	[27]
	*Labeo rohita*	Rohu carp	FW		CT (D)	I	-	18	LOE	26–36	12.0–14.4	42.3–45.6	[28]
	*Pimephales promelas*	Fathead minnow	FW	3 dph	CT (D)	G	-	18	LOE	22–23	3.4–9.9	31.4–35.9	[29]
Ord. Cyprinodontiformes													
Fam. Cyprinodontidae													
	*Cyprinodon nevadensis*	Amargosa pupfish	FW	60 dph	CT (D)	G	-	18	LOE	20–36	-	38.0–44.0	[30]
Fam. Fundulidae													
	*Fundulus grandis*	Gulf killifish	SW	<9 mm SL	CT (D)	I	-	18	LOE	29	-	42.6–43.6	[31]
	*Fundulus heteroclitus*	Mummichog	SW	<9 mm SL	CT (D)	I	-	18	LOE	29	-	42.8–44.5	[31]
Ord. Esociformes													
Fam. Esocidae													
	*Esox lucius*	Northern pike	FW	YS	LT (S)	G	24.0–168.0	abrupt change	D	6–18	-	20.4–28.9	[32]
			FW	YS	LT (S)	-	240.0	3	D	3–24	4.2	> 25.0	[33]
	*Esox masquinongy*	Muskellunge	FW	1–53 dph	CT (D)	G	1272.0	60	S	17–23	-	29.0–35.0	[34]
Ord. Gadiformes													
Fam. Gadidae													
	*Gadus morhua*	Atlantic cod	SW	E to YS	LT (S)	G	24.0–600.0	-	D	6	-	>12	[35]
Ord. Mugiliformes													
Fam. Mugilidae													
	*Mugil cephalus*	Hawaiian striped mullet	SW	YS	LT (S)	G	168.0	2		3–33	14.2^a^	30.1	[36]
Ord. Osmeriformes													
Fam. Osmeridae													
	*Hypomesus transpacificus*	Delta smelt	FW	30–64 dph	CT (D)	I	-	18	LOE	16	-	29.0–30.0	[37]
	*Mallotus villosus*	Capelin	SW	2–4 dph	LT (S)	G	24.0 (+ 0.3)	-	D	5	-2.0–-3.0	> 20.0	[38]
	*Osmerus mordax*	Rainbow smelt	FW	-	LT (S)	G	0.1–1.0	-	D	13	-	29.0–32.0	[21]
Ord. Perciformes													
Fam. Carangidae													
	*Atule mate*	Yellowtail scad	SW	0–6 dph	LT + ILT (S)	G	0.5–72.0	-	D	24	-	26.0–37.0	[39]
Fam. Centrarchidae													
	*Micropterus salmoides*	Largemouth bass	FW	0–12 dph	LT (S)	G	24.0	-	D	20–30	-	31.2–33.7	[40]
			FW	YS, FL	LT (S)	G	1.0–96.0	0–5	D	18–38	-	32.8–34.1	[41]
Fam. Cichlidae													
	*Oreochromis mossambicus*	Mozambique tilapia	FW	E to YS	LT (S)	G	240.0	-	D	11–40	20.0	> 34.0	[42]
	*Oreochromis niloticus*	Nile tilapia	FW	YS	LT (S)	G	YS to swim-up larvae	-	D	11–40	21.8	32.1	[43]
			FW	13–32 mm TL	CT (D)	-	24.0–60.0	0.04	D	25–37	-	38.0–39.0	[44]
Fam. Moronidae													
	*Morone chrysops*	White bass	FW	1 dph	LT (S)	G	24.0	-	D	14–26	-	30.8–32.0	[45]
	*Morone saxatilis*	Striped bass	FW	3–14 mm TL	LT (S)	G	24.0	abrupt	D	15–23	-	31.7–36.7	[46]
Fam. Percidae													
	*Etheostoma fonticola*	Fountain darter	FW	24–72 hph	LT (S)	G	24.0	-	D	23	3.8	-	[47]
	*Perca flavescens*	Yellow perch	FW	E to YS	LT (S)	-	YS to swim-up larvae	-	D	12	9.3–9.8	18.8–22.5	[48]
			FW	YS	LT (S)	-	24.0	-	D	18	3.0	28.0	[49]
	*Sander lucioperca*	Pikeperch	FW	4–6 mm	LT (S)				D		6.0–6.5	30.0–32.0	[50] in [49]
Fam. Sciaenidae													
	*Bairdiella icistia*	Bairdiella	SW	2 mm	LT (S)	G	1.0–72.0	abrupt	D	21–30	-	29.0–36.0	[51]
Fam. Scombridae													
	*Thunnus albacares*	Yellowfin tunna	SW	YS	LT + ILT (S)	G	E to YS	0.04	M + D	19–36	19.5	35.2	[52]
Fam. Sparidae													
	*Pagrus major*	Red sea bream	SW	0–42 dph	LT (S)	-	24.0	-	D	20	9.5–12.0	26.5–30.5	[53]
	*Sparus aurata*	Gilt-head bream	SW	12 dph	CT (D)	G	-	1	SWI	18	-	22.0–30.0	[54]
Ord. Petromyzontiformes													
Fam. Petromyzontidae													
	*Ichthyomyzon fossor*	Northern brook lamprey	FW	AMM	LT (S)	-	336.0	-	D	15	-	30.5	[55]
	*Lampetra lamottenii*	Brook lamprey	FW	AMM	LT (S)	-	336.0	-	D	15	-	28.5	[55]
	*Lampetra planeri*	European brook lamprey	FW	AMM	LT (S)	-	336.0	-	D	5–25	-	27.0–29.0	[55]
	*Petromyzon marinus*	Sea lamprey	FW	AMM	LT (S)	-	336.0	-	D	5–25	-	29.5–31.0	[55]
Ord. Pleuronectiformes													
Fam. Pleuronectidae													
	*Pleuronectes putnami*	Smooth flounder	SW	-	LT (S)	G	0.1–1.0	-	D	4	-	27.0–32.0	[21]
	*Pseudopleuronectes americanus*	Winter flounder	SW	5 dph	LT (S)	G	0.1–1.0 (+ 24.0)	-	D	5	-	29.0–33.0	[56]
Fam. Scophthalmidae													
	*Scophthalmus maximus*	Turbot	SW	0–25 dph	LT (S)	G	2.0 (+ 60.0–84.0)	-	D	17	-	22.0–29.0	[57]
Fam. Soleidae													
	*Solea solea*	Dover sole	SW	1–12 mg WW	LT (S)	G	96.0	-	D	8–15	5.0–8.7	23.0–28.1	[58]
Ord. Salmoniformes													
Fam. Salmonidae													
	*Coregonus artedi*	Cisco	FW	-	LT (S)	G	24.0	-	D	3	-	19.8	[59]
	*Oncorhynchus clarkii virginalis*	Cutthroat trout	FW	7–14 dph	LT + ILT (S)	G	720.0–1440.0	0.04	D	10–26	-	22.6–25.7	[60]
	*Oncorhynchus gilae apache*	Apache trout	FW	E to YS	LT (S)	G	336.0	0.25	D	15–27	-	17.1–17.9	[61]
	*Oncorhynchus kisutch*	Coho salmon	FW	E to YS	LT (S)	G	> 1400.0	-	D	1–17	-	12.5	[62]
	*Prosopium williamsoni*	Mountain whitefish	FW	30–300 mg WW	LT + ILT (S)	I	0.1–792.0	0.04	D	10	-	22.6–23.6	[63]
			FW	870–3700 mg WW	CT (D)	I	-	30	LOE	13	-	26.7	[63]
	*Salmo salar*	Atlantic salmon	FW	-	LT (S)	G	120.0–168.0	1	D	5–6	-	22.0–28.0	[64]
	*Salmo trutta fario*	Brown trout	FW	-	LT (S)	G	120.0–168.0	1	D	5–6	-	23.0–28.0	[64]
	*Salmo trutta trutta*	Sea trout	FW	-	LT (S)	G	120.0–168.0	1	D	5–6	-	22.0–28.0	[64]
	*Salvelinus alpinus*	Arctic charr	FW	13–15 mm	LT + ILT (S)	G	72.0 (+ 0.2–168.0)	2	D	0–20	-	19.3–26.2	[65]
Ord. Scorpaeniformes													
Fam. Sebastidae													
	*Sebastes thompsoni*	Rockfish	SW	-	LT (S)	-	24.0	-	D	10–25		25.6–28.8	[66]
Ord. Siluriformes													
Fam. Clariidae													
	*Clarias gariepinus*	African sharp-tooth catfish	FW	E to YS	LT (S)	G	>193.0	-	D	17–36	18.9	33.2	[67]

Abbreviations: FW, freshwater; SW, seawater; LT, lethal temperature; ILT, incipient lethal temperature; CT, critical temperature; S, static method; D, dynamic method; dph, days post-hatch; E, eggs; YS, yolk sac larvae; FL, feeding larvae; AMM, ammocoetes; TL, total length; SL, standard length; WW, wet weight; I, individuals; G, groups; D, death; LOE, loss of equilibrium; S, onset of spasms; M, malformations; SWI, swimming ceases.

Upper thermal limits in ectotherms have been estimated using either static or dynamic methods [14,68]. The former exposes groups of fish to different, constant temperatures (exposure time varies) to estimate the temperature at which 50% of the individuals in the group die which, depending on exposure time, has been referred to as the Upper Lethal Temperature (*LT50*_max_) [20,22] or Upper Incipient Lethal Temperature (UILT, *sensu* Fry, [69]) [39]. On the other hand, the dynamic method exposes individuals or groups of fish to a constant increase in temperature (starting at the ambient temperature) until physiological failure is noted (e.g. muscular spasms, loss of equilibrium, motor function stops) [14]. The Critical Thermal Maximum (*CT*_*max*_) is estimated using the dynamic method and it is defined as “the thermal point at which locomotory activity becomes disorganized and the animal loses its ability to escape from conditions that will promptly lead to its death” [14, p.1562]. Since the 1980s, *CT*_*max*_ has been estimated more frequently than *LT50*_max_, which is likely due to the fact that the former is easier to apply, it requires fewer animals and takes less time compared to the latter [14]. Also, *CT*_*max*_ is considered to be more ecologically relevant than *LT50*_max_, as it sets the upper limit in thermal reaction norms (or performance curves), and uses (or can use) warming rates observed *in situ* [70,71]. Nevertheless, lethal (*LT50*_*min*_ / *LT50*_*max*_) as opposed to critical (*CT*_*min*_ / *CT*_*max*_) thermal limit protocols have been and continue to be used in most studies performed on larval fish [15,61], although the number of studies using the dynamic method has markedly increased since 2010 (Fig 1).

Small differences in the protocol used may have large impacts on *CT*_*max*_ [70,72,73], as occurs with other time-dependent tolerance measurements (e.g critical swimming speed, hypoxia tolerance) [74,75]. Warming rate is likely the most sensitive parameter in the *CT*_*max*_ protocol. Previous protocols have used warming rates between ~0.1°C min^-1^ to 0.1°C h^-1^ and the choice is not trivial: faster rates than those generally experienced by the organism in the wild (e.g. diurnal differences in temperature) may overestimate *CT*_*max*_ due to an impairment of physiological processes acting on the organism, whereas slower rates may underestimate *CT*_*max*_ due to longer exposures to warm temperatures and accumulation of heat damage [70,73,76]. Other studies have pointed out, however, that very slow warming rates may allow animals to acclimate to warmer temperatures, which would lead to an overestimation of *CT*_*max*_ [77]. Such slow heating rates (e.g. 1°C h^-1^, 1°C d^-1^, 2.5°C week^-1^) have been employed to explore the adaptive capacity of thermal tolerance such as work by Morley et al. [78] comparing invertebrates from different latitudes. Therefore, the choice of the *CT*_*max*_ protocol and the corresponding interpretation of the results and application to field conditions needs to be done with care.

Most studies of *CT*_*max*_ (or *CT*_*min*_) in fish larvae have used a warming or cooling rate of 0.3°C min^-1^ [27,37,63], a rate recommended in protocols established for juvenile and adult fish by Becker and Genoway [13]. However, the impact of different warming rates on *CT*_*max*_ of marine fish larvae has never been assessed. Understanding the impact of methodology on the estimation of critical thermal limits is essential to develop methods that take into account the specific traits of early stages of fish (e.g. higher surface-to-volume ratio, handling sensitivity, greater sensitivity to starvation).

We investigated the upper thermal tolerance (*CT*_*max*_) of larvae of Atlantic herring (*Clupea harengus*) and European seabass (*Dicentrarchus labrax*). We explored how *CT*_*max*_ was influenced by developmental stage, acclimation temperature as well as warming rate. To our knowledge, this is the first study to examine how methods used in protocols affect estimates of *CT*_*max*_ in fish larvae. Recommendations for developing protocols to estimate critical thermal maxima and minima in larval fish are also provided.

## Materials and methods

### Ethics

Experiments on Atlantic herring were performed under the German law on experimental animals and were approved by the Ethics Committee of the Hamburg Authority for Health and Consumer Protection (Application nr. 95/11). Those on European seabass were performed under French national regulations and approved by the Comité d’Éthique Finistérien en Expérimentation Animale (CEFEA- registering code C2EA–74) (Autorisation APAFIS 4341# 2016030214474531).

### Herring larval rearing

Adult herring were obtained from a commercial fisherman in the Kiel Fjord (54.36°N, 10.13°E) in March 2014, and transported on ice to the Elbe Aquarium of the Institute of Hydrobiology and Fisheries Science, University of Hamburg. Eggs were strip-spawned from 21 females (mean (± SD) length, 24.7 (± 1.1) cm; mean (± SD) wet weight, 167.1 (± 30.2) g) and fertilized using the milt from 10 males (mean (± SD) length, 24.6 (± 1.0) cm; mean (± SD) wet weight, 168.1 (± 27.9) g). The large number of females and males helped avoid any parental effects on offspring quality. Egg and larval rearing conditions were similar to those described in Moyano et al. [79], except for the rearing temperatures. Briefly, dark green, circular 90-L tanks containing filtered seawater (0.5 μm, Reiser Filtertechnik GmbH, Seligenstadt am Main, Germany) renewed at 50% d^-1^ were used to incubate eggs and rear larvae. Temperature was measured every 10 minutes (TLog64-USB, Hygrosens, Donaueschingen, Germany) and salinity (WTW cond3110 probe, Weilheim, Germany) and ammonium (Tetra NH_3_/NH_4_^+^ kit, Spectrum Brands, VA, USA) were measured daily. The light regime was 14 L: 10 D. Eggs were incubated at a mean (± SD) temperature of 9.0 (± 0.4°C, and salinity of 16.2 (± 0.5). After hatching, *ca*. 1600 larvae were transferred to the new rearing tanks, and temperature was adjusted 0.5°C d^-1^ to a rearing temperature of either 7 or 13°C, with two replicate tanks (A and B) at each temperature. The final temperature was reached at a larval age of 9 days post-hatch (dph), after which the mean (± SD) temperature was 7.6 (± 0.4), 7.5 (± 0.4), 12.7 (± 0.2), and 12.8 (± 0.3) °C in tank 7A, 7B, 13A and 13B, respectively. The mean salinity of each tank was between 16.3 (± 0.4) and 16.6 (± 0.5). Larvae were reared in the presence of algae (*Rhodomonas baltica*, 10,000 cell mL^-1^) and dinoflagellates (*Oxyrrhis marina*, 1,000 cells mL^-1^) and fed natural prey (different stages of the copepod *Acartia tonsa*), supplemented with small amounts of brine shrimp (*Artemia sp*.) nauplii.

Each week, 20 larvae were taken out from each rearing tank to obtain length and weight estimates for growth rate calculations. They were anesthetized with metomidate (10 mg L^-1^, Aquacalm, Syndel Laboratories, BC, Canada), digitally photographed under a stereomicroscope (Leica MZ 16, Wetzlar, Germany), euthanized with an anesthetic overdose and stored at -80°C. Body length (measured as notochord length for preflexion larvae and standard length for flexion and postflexion larvae) was measured using ImageJ [80]. Finally, larvae were freeze-dried (0.200 mbar; >16 h, Christ Alpha 1–4 LSC, Osterode am Harz, Germany) and weighed (± 0.1 μg, Sartorius Genius SE2 microbalance, Göttingen, Germany).

### Seabass larval rearing

Three-day old seabass larvae were obtained in January 2016 from Aquastream, a commercial hatchery in Ploemeur (France). These larvae were the progeny of wild spawners (Morbihan, France) including four females (mean weight 4.5 kg) and ten males (2.4 kg). Spawners were maintained at 13°C, with a light regime of 8.75 L: 15.25 D, and a water salinity of 35.

After larvae were transported to Ifremer-Centre de Bretagne, *ca*. 5000 were distributed to each of three grey, 35-L tanks located in a temperature-controlled room. Water temperature, salinity, pH and dissolved oxygen concentration were monitored daily (WTW Multi3410, Weilheim, Germany). Mean (± SD) temperature and salinity was 20 (± 0.1) °C and 33 (± 0.2), respectively. A light regime of 15 L: 9 D was used, and the light intensity gradually increased from 0 lux (3 dph) to 96 lux (44 dph). Larvae were fed brine shrimp (*Artemia* sp) nauplii *ad libitum* using automatic feeders.

Every 8–10 days, 20 larvae were collected from each tank, anesthetized with MS-222 (Tricaine Methane Sulfonate 1000 mg g^-1^, PHARMAQ, Hampshire, UK), photographed and stored at -80°C to measure body length and dry weight, using the same equipment and procedures as for herring larvae.

### *CT*_*max*_ trials

A total of eight *CT*_*max*_ trials was conducted on herring larvae and two on seabass larvae (Table 2). Each *CT*_*max*_ trial used four thermal control units (Fisherbrand FBC 30, Fisher Scientific GmbH, Schwerte, Germany) which contained nine 250-mL beakers. One unit was used as a control and maintained at the starting temperature throughout the trial (max. 40 h for herring, 10 h for seabass). Warming rate treatments were randomly assigned to a thermal control unit in each trial. At the start of each trial, the water temperature and salinity and the light regime were the same as those experienced by larva in their rearing tank. Each beaker was aerated using a small pump (Tetra APS400, Spectrum Brands, VA, USA) with small bubbles produced using a fine glass pipette. Individual larvae were randomly collected from replicate rearing tanks at the test temperature and gently transferred to a beaker. Beakers were randomly assigned to a treatment group (warming rate). For seabass, 3 larvae were randomly collected from each of the three replicate tanks for each warming rate (9 individuals in total). After a 15-min acclimation period, the *CT*_*max*_ protocol was started. In total, five different warming rates were examined for herring (0.5, 1, 2, 4, and 8°C h^-1^) and seabass (1.5, 3, 6, 6 + 1.5, and 9°C h^-1^). The heating rate “6 + 1.5” for seabass consisted of a quick start (6°C h^-1^) up to a point close to the expected *CT*_*max*_ (i.e. ~ 26°C), followed by a slower heating rate (1.5°C h^-1^). Laboratory access regulations required that all work on seabass be completed in <9h. For all slow warming treatments (or controls) which lasted >14h (i.e. 0.5, 1°C h^-1^ in herring), constant light (24 L: 0 D) was used. No prey was added to the beakers at any point during a trial.

**Table 2:** Details of the Critical Thermal maxima (*CT*_*max*_) trials conducted with Atlantic herring and European seabass larvae. Note “age” refers to the days-post hatch at the start of the *CT*_*max*_ trial, and “size” is the mean larval size of all the larvae used in each *CT*_*max*_ trial.

Species	Trial Nr.	Age (dph)	Age (dd)	Size (mm, mean ± SE)	Rearing T (°C)	Warming rate (°C h^-1^)	n*
Herring	1	1	9	7.8 ± 0.1	9	0.5, 1, 2, 4, 8	9
	2	20	140	10.3 ± 0.2	7	0.5, 1, 2, 4, 8	9
	3	45	327	14.4 ± 0.5	7	1, 2	9
	4	55	402	17.1 ± 0.3	7	0.5, 1, 2, 4, 8	9
	5	66	491	20.9 ± 0.3	7	1, 2	9
	6	14	130	12.8 ± 0.2	13	0.5, 1, 2, 4, 8	9
	7	34	387	21.2 ± 0.3	13	0.5, 1, 2, 4, 8	9
	8	43	499	21.2 ± 0.3	13	1, 2	9
Seabass	9	15–16	283	6.5 ± 0.1	20	1.5, 3, 6, 6+1.5, 9	9
	10	43–44	863	14.1 ± 0.2	20	1.5, 3, 6, 6+1.5, 9	9

Abbreviations: dph, days post-hatch; dd, degree-days; T, temperature; SE, standard error.

* *n* per trial (warming rate).

Once the warming protocol started, larvae were checked every 15 min (only every 30 min between the hrs of 23:00 and 06:00 for the 0.5 and 1°C h^-1^ rates used in herring), and the state of the larva and the temperature was recorded (P700, ±0.1°C, Dostmann electronic, Wertheim-Reicholzheim, Germany). The *CT*_*max*_ endpoint was considered to be the loss of equilibrium. Once a larva had lost its equilibrium, it was taken out of the beaker, anesthetized with metomidate (10 mg L^-1^), digitally photographed under a stereomicroscope, euthanized by an anesthetic overdose and stored at -80°C. Body length and dry weight were measured using methods previously described.

### Statistical analysis

Herring specific growth rates (dry weight, % *SGR*_*DW*_) were calculated between 15–66 dph for rearing tanks 7A and 7B, and 14–45 dph for 13A and 13B. For seabass, *SGR*_*DW*_ was calculated between the ages of 17 and 46 dph.

The effect of warming rate and body length on *CT*_*max*_ was assessed using generalized linear models [81]. Three different models were used, one for yolk-sac herring larvae, one for exogenously feeding herring larvae and one for exogenously feeding seabass larvae. GLMs included warming rate, body length and acclimation temperature (if present) as fixed effects for herring and seabass exogenously feeding larvae. In order to avoid heteroscedasticity, different variances were allowed depending on body length per warming rate treatment. In the case of herring yolk sac larvae, body length was not included as a fixed effect (all larvae were similar in size). A backward model selection procedure starting with the most complex (i.e. interactive effect between all fixed factors) and ending with all non-significant factors removed [82] helped identify which variables influenced *CT*_*max*_. The residuals of each final model were plotted against all significant predictors to identify any remaining heteroscedasticity and check that no relationships between predictors and *CT*_*max*_ were ignored. In addition, the model residuals were also checked for a potential effect of rearing tank by analysis of variance (ANOVA).

Previously published estimates of *LT50*_max_ for herring larvae were combined with the *CT*_*max*_ data collected in this study to explore the time dependency of upper thermal limits. The arithmetic mean value of *CT*_*max*_ for each treatment group in each trial was calculated and the upper thermal limits were plotted against the total time larvae were exposed to temperatures > 18°C. This temperature threshold was selected as a proxy for pejus temperature (*T*_*pej*_) in the light of temperature-dependent growth rates of herring larvae [83].

All analyses were carried out using the R statistical software [84] with the nlme package [81].

## Results

Herring larvae hatched 12 days after fertilization with a mean (± SE) body length of 7.9 (± 0.2) mm. The mean *SGR*_*DW*_ after yolk sac absorption was 4.7, 3.5, 9.3 and 14.0% d^-1^ for larvae in tanks 7A, 7B, 13A and 13B, respectively.

Seabass larvae reared at 20°C had a mean *SGR*_*DW*_ between 10.3 and 13.9% d^-1^ in the different rearing tanks.

### *CT*_*max*_ in herring larvae

**Fig 2:**
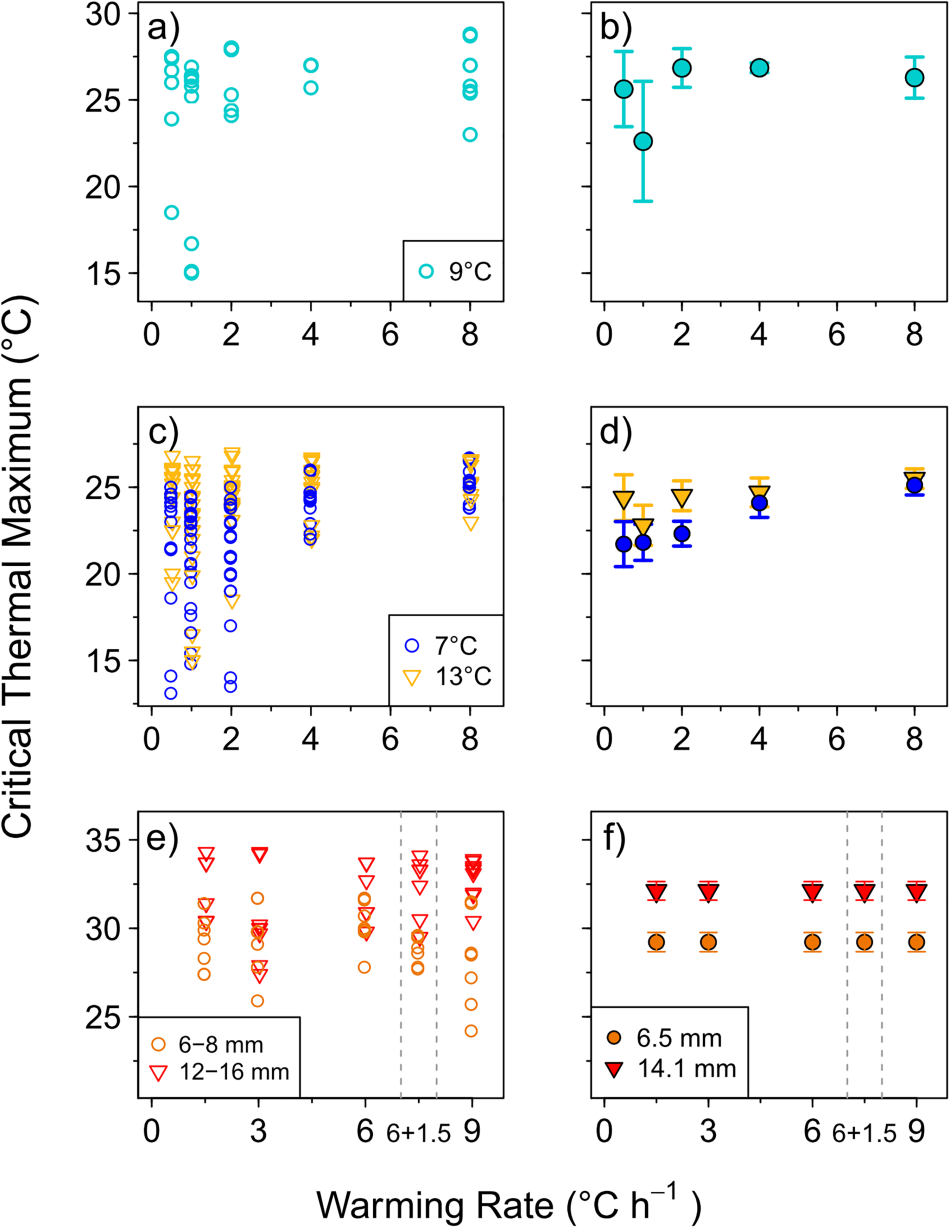
**Critical thermal maxima (*CT*_*max*_) estimates of Atlantic herring yolk sac larvae (a-b) and exogenously feeding larvae (c-d), and European seabass exogenously feeding larvae (e-f) at different warming rates.** Left-hand panels show *CT*_*max*_ of individual larvae. Right-hand panels show the mean treatment values (± 95% CI) from Generalized Linear Model (see S1 Table), except for yolk sac larvae (panel b) in which mean (±95% CI) *CT*_*max*_ values are shown (as no model was fitted to this dataset).

Larval survival in the controls was generally 89–100% (a maximum of 1 larva died during a trial) except in trial 2 (7°C, 140 degree-days) where survival was 67% (Table 2). The *CT*_*max*_ value varied markedly among individuals (range 13°C), especially among larvae experiencing slower warming rates (≤ 2°C h^-1^). In yolk sac larvae, *CT*_*max*_ ranged from 15.0 to 28.8°C and treatment means were between 22.6 and 26.8°C (Fig 2A). Warming rate had no significant effect on *CT*_*max*_ of yolk sac larvae (GLM, not shown). For exogenously feeding larvae, *CT*_*max*_ values ranged from 13.1 to 27.0°C (Fig 2C) and faster warming rates (4 and 8°C h^-1^) led to significantly higher *CT*_*max*_ values compared to slower rates (0.5, 1, and 2°C h^-1^) (p< 0.05) (Fig 2D, S1 Table). Overall, *CT*_*max*_ was significantly warmer for larvae reared at 13°C compared to 7°C (increase of 2.7 ± 0.9°C, S1 Table) but the differences in *CT*_*max*_ between acclimation temperatures were relatively minor (~0.7 and 0.4°C) for larvae in faster (4 and 8°C h^-1^) warming rate treatments. Body length had no significant effect on *CT*_*max*_ in exogenously feeding herring larvae (S1 Fig) nor did rearing tank (ANOVA, p>0.05).

### *CT*_*max*_ in seabass larvae

The *CT*_*max*_ of individual larvae ranged from 24.2 to 34.3°C with treatment-specific means between 27.8 and 32.8°C (Fig 2E). Warming rate had no significant effect on *CT*_*max*_, (p = 0.505) but body length had a highly significant effect (p < 0.01) (Fig 2F, S1 Table). Overall, larger larvae (14.1 mm mean size) had a significantly higher *CT*_*max*_ than smaller larvae (6.5 mm mean size). Rearing tank had no effect on *CT*_*max*_ (ANOVA, p>0.05).

### Time dependency of thermal tolerance

**Fig 3:**
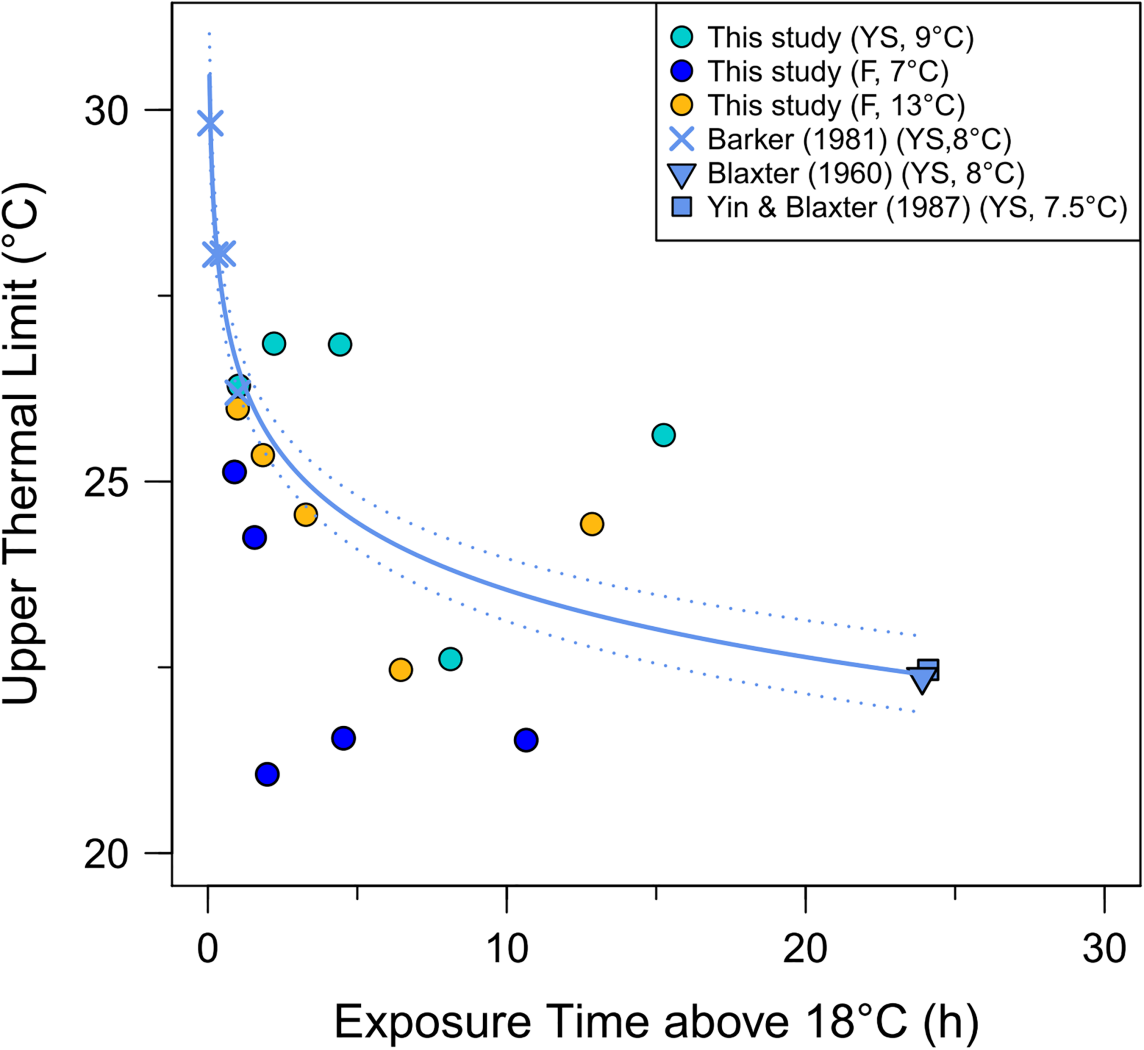
Time dependency of upper thermal limits in Atlantic herring larvae. Values for upper thermal limit (*UTL*, °C) including both *LT50*_max_ and *CT*_*max*_ estimates (see text) versus exposure time (*t*, h) beyond temperatures favorable for growth (>18°C). The *LT*_*max*_ and *CT*_*max*_ estimates for yolk-sac (YS), and feeding larvae (F) at two temperatures (7 and 13°C) are also shown. For the *LT50*_*max*_ data of YS larvae, the best fit regression equation (solid line) is *UTL* = 26.55(± 0.16 SE)—1.31(±0.08 SE) * Ln(*t*), (p<0.001), 95% CI of the curve are included as a dotted line.

The data from this and previous studies on herring yolk sac larvae suggest that survival time decreases with increasing exposure time above *T*_*pej*_ (18°C) according to a negative, logistical model (Fig 3). When exposed to temperatures > *T*_*pej*_ for 30min, *LT50*_max_ was ~28–30°C whereas *LT50*_max_ declined to 22.5°C after a 24-h exposure period. Similarly, for exogenously feeding larvae reared at both 7 and 13°C, shorter exposure times (in faster warming rate treatments) were associated with higher mean *CT*_*max*_ (Fig 3). However, this time-dependency of *CT*_*max*_ was not evident for yolk sac larvae from this study.

## Discussion

Obtaining robust estimates of thermal tolerance in ectotherms is fundamental if we hope to make projections of species performance under future climate scenarios. Early life stages of fish are expected to have a narrower thermal tolerance than juveniles and adults [9,12]. However, relatively few studies have been published on thermal limits of fish larvae, especially for marine species (Table 1). The lack of a standard methodology for larvae may be slowing our progress to compile data which can be compared across life stages and species, as has been done with other larval traits (e.g. critical swimming speed, [85]). Here we explored the impact of, arguably, the most important methodological factor (warming rate) on the upper thermal limit of the larvae of two temperate marine species. Using these results, together with a compilation from previous studies on thermal tolerance in fish larvae, we make recommendations for protocols to be used to estimate thermal limits in temperate marine larvae. Further tests in other groups (e.g. polar, tropical species) might enable “a universal protocol” to be developed for larval fish, which would facilitate intra- and interspecific comparisons.

### Upper thermal limits in Atlantic herring and European seabass

The *CT*_max_ values measured here for yolk sac larvae of herring (treatment means 22–27°C) are in the range of the lethal temperatures previously estimated for this species and life stage [20–22]. The *CT*_max_ was slightly lower (treatment means 21–26°C) in exogenously feeding larvae, and a similar decline in *LT50*_max_ was observed by Yin & Blaxter [20] when comparing larvae prior to and after yolk sac absorption. However, *CT*_*max*_ did not significantly differ across body sizes in exogenously feeding larvae. These *CT*_*max*_ values agree well with the life cycle scheduling of Atlantic herring in the southwest Baltic Sea, where herring start spawning in coastal areas in early spring after temperatures increase above 5°C. Waves of spawning occur producing larvae that inhabit coastal nurseries until early summer when temperatures are 15 to 20°C [86]. Then large larvae (> 20 mm) migrate to deeper, colder offshore waters which represent the feeding grounds of juveniles and adults. Although far below *CT*_max_, chronic (long-term) exposure to temperatures above upper *T*_*pej*_ (~18°C) may impact growth performance and, ultimately, survival. It should also be noted that cold snaps (5 to 2.5°C) in early spring can lead to massive mortalities as reported for larval herring in the Vistula Lagoon in the southern Baltic Sea [87].

European seabass larvae could tolerate warmer temperatures (*CT*_*max*_ treatment means 28–33°C) than Atlantic herring larvae. In contrast to herring, thermal tolerance of seabass appeared to increase with increasing size and this may continue into the juvenile stage where *CT*_*max*_ values of ~ 28–35°C have been reported [88]. This increase in thermal tolerance with size/age matches the migratory life cycle for this species [89]. In the North Atlantic and Mediterranean Sea, spawning occurs offshore in winter (8.5–15°C for the southern North Sea, [90]). Post-larvae enter shallow, sheltered coastal estuaries or lagoons in spring, where they remain as juveniles during the warm summer period when they are likely exposed to warm snaps of >28°C [91].

**Fig 4:**
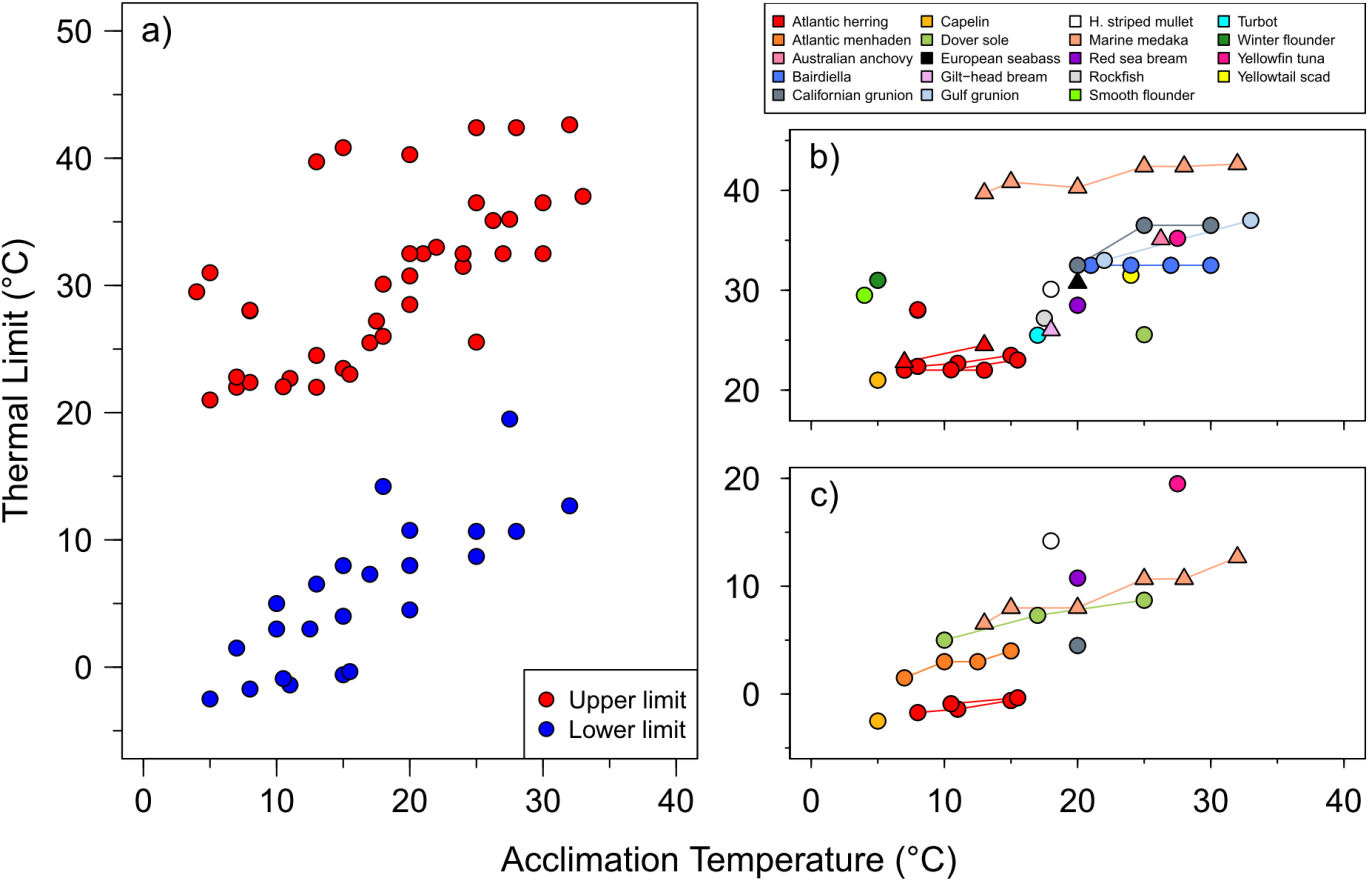
Upper and lower thermal limits of marine fish larvae. a) Average upper (red) and lower thermal limits (blue) of marine fish larvae at different acclimation temperatures. b) Detail of the upper (*LT50*_*max*_, *CT*_*max*_), and c) lower limits (*LT50*_*min*_, *CT*_*min*_), color-coded by species and shape-coded by method (static, circles; dynamic, triangles). Lines connect estimates from the same study. Study details are provided in Table 1.

The *CT*_*max*_ of herring larvae is in the range of the *CT*_*max*_ or *LT50*_*max*_ of larvae from other cold-temperate marine species such as capelin (*Mallotus villosus*) or turbot (*Scophthalmus maximus*) acclimated to similar temperatures (Fig 4). The *CT*_*max*_ values for seabass larvae are warmer and closer to *LT50*_*max*_ estimates for red sea bream (*Pagrus major*) and Hawaiian striped mullet (*Mugil cephalus*). But these upper thermal limits for herring and seabass are much colder than, for example, those estimated for medaka (*Oryzias melagstima*) (*LT50*_*max*_ > 40°C) reared at the same acclimation temperature. The available estimates of both the lower and upper thermal limits of a small number of species suggest that larvae of some species (e.g. herring or capelin) have a wider thermal tolerance than others (e.g. Dover sole *Solea solea*). Unfortunately, these comparisons need to be taken *cum grano salis*, as different methods were applied. In studies of lethal limits, a variety of exposure times (from minutes to weeks) and transfer rates to new temperatures (acute change or acclimation allowed) were used. In studies of *CT*_max_, different rates of warming (or cooling) have been applied (Table 1). With the present data compilation and our measurements of the effect of warming rate on thermal limits, we hope to stimulate the community to create a standard protocol for early life stages of fish.

### Impact of methodology on *CT*_*max*_ estimates

Although estimating *CT*_*max*_ is one of the most common methods used to assess upper thermal limits in ectotherms such as fish, surprisingly, no general consensus exists on the protocol. The same is true for protocols developed to measure other physiological traits such as critical swimming speed [74] and hypoxia tolerance [75]. Hence, the community is often faced with a broad range of values obtained with a large variety of methodologies. Thus, a disjunctive situation arises in which some employ a standard protocol (for example, to make intra- or interspecific comparisons), while others use protocols adjusted to a specific research objective or to a particular environmental event of condition being examined. The first *CT*_*max*_ protocols developed for juvenile and adult fish employed fast, arguably unrealistically fast, rates of temperature change, 18°C h^-1^ [13], 60°C h^-1^ [14]. On the other hand, more recent *CT*_max_ protocols have been tailored to the research question addressed, e.g. tolerance to heat waves in enclosed bays (1°C h^-1^, [92]), long-term adaptation of heat tolerance in relation to global warming using much slower “ecologically relevant” rates (from 1°C d^-1^ to 1°C month^-1^, [93]) or *CT*_max_ as a measurement of fish health in challenge tests (7 + 0.5°C h^-1^, [94]). Recently published *CT*_*max*_ studies on fish larvae have used warming rates ≥ 0.3°C min^-1^ (18°C h^-1^) [31,37] (Table 1), regardless of whether the aim was to estimate upper thermal tolerance for aquaculture or biogeographical distribution. Surprisingly, the present study is the first (to the best knowledge) to try to reconcile the different options available (e.g. standardization vs tailoring) by examining the impact of warming rate on *CT*_*max*_ in larval fish. Our results show clear, species-specific effects of warming rate on larval *CT*_*max*_ as suggested for adult fish and other taxa (e.g. Crustacea) [72,95,96].

In this study, Atlantic herring exposed to warming rates ≤ 2°C h^-1^ had lower *CT*_*max*_ values than those exposed to faster rates, likely due to oxygen limitation and accumulation of anaerobic end products [97]. At the slowest warming rates, thus, no acclimation potential was observed, suggesting that even slower rates would be needed to explore thermal acclimation (e.g. 1°C d^-1^) [78]. Within the slow warming rate treatments applied here, *CT*_*max*_ was much more variable among individuals, especially for 7°C-reared larvae, probably due to the relatively long exposure time to warmer temperatures (i.e. using a warming rate of 1°C h^-1^, reaching a *CT*_*max*_ of 27°C takes 20h from 7°C, and 14h from 13°C). Interestingly, patterns of inter-individual variation suggest that slower warming rates may help one better distinguish larvae with different traits or condition compared to faster warming rates. The pattern of change in *CT*_*max*_ versus warming rate observed here agrees well with the typical trends observed in other ectotherms [70,73,76]. Additionally, no effect of rearing tank, and thus growth rate, was observed. In the case of herring yolk sac larvae, warming rate had no effect on *CT*_*max*_. This discrepancy in the trends observed for yolk sac larvae and for later larval stages may be related to the low number of yolk sac larvae tested (i.e. only one trial was conducted, *n* = 9 per warming rate), and the high inter-individual variability in *CT*_*max*_ observed at the slow warming rates. Given the variation observed here, a minimum sampling size of 15–20 individuals per trial and/or warming rate would be recommended. Moreover, it is also more challenging to assess the loss of equilibrium in very small, slow-moving larvae, thus, some authors have used cessation of swimming as a *CT*_*max*_ endpoint [54].

In seabass larvae, warming rate had no effect on *CT*_*max*_ for a given larval size. This pattern could be due to some degree of adaptation potential in heat tolerance at the slowest warming rates, as was suggested for other ectothermic species [76]. Alternatively, it could be due to the relatively narrow range of warming rates tested (1.5 to 9.0°C day^-1^), since, in other species, *CT*_*max*_ was only significantly reduced at warming rates <1°C h^-1^ [96 and references herein]. Additionally, no significant differences were found (in the mean or variance in *CT*_max_) between constant warming rates (e.g. 6°C h^-1^) and variable rates that combine a fast start (6°C h^-1^) with a slower rate afterwards (1.5°C h^-1^). This latter method has been used for adult fish, and is thought to better resolve potential inter-individual differences in *CT*_*max*_ [98]. Combining fast and slow heating rates appears to be a good method to use because it decreases the duration of *CT*_max_ trials but still makes it practical to ascertain the exact endpoint temperature.

Estimating thermal tolerance in early life stages of fish presents unique challenges, e.g. larvae can have high growth rates (*ca*. 10–30% d^-1^) and have, in general, a low resistance to starvation. Long trials using very slow warming rates (e.g. 1°C d^-1^) could yield *CT*_*max*_ estimates, which may be difficult to interpret as larvae pass through different developmental stages (leading to an “integrated *CT*_*max*_”), or may be biased by other time-dependent processes such as starvation [20,99]. If one aims to identify differences across developmental stages, we argue that a measurement of an “instantaneous *CT*_*max*_”, estimated over one day, is most appropriate. For example, estimates from such rapid methods can be compared across life stages to identify bottlenecks in the persistence of populations in warmer waters in the future (e.g. [11,37]). Since one cannot predict, *a priori*, how the warming rate will impact on the *CT*_*max*_ of the larvae of different fish species using one standard heating rate across species is likely unwarranted [95,96]. In this context, we recommend that different heating rates (e.g. from 1 to 10°C h^-1^) be tested in pre-trials and that one chooses a rate associated with the highest *CT*_max_, as has been proposed for adult fish [96]. For other contexts, such as using *CT*_max_ as an indicator of fish health against several stressors, one could choose a rate that is more practical (logistically) for the study and compare the estimates across treatments.

Several studies have highlighted the relationship between *CT*_*max*_ and the geographic range of a species and/or population [9,100], and the importance of quantifying the difference between *CT*_*max*_ and habitat temperature in order to explore the likelihood that this thermal buffer is exceeded during warm episodes [101,102]. In the light of this research, one could argue that the generation of a standard protocol for estimating what we have termed the “instantaneous *CT*_*max*_” would allow intra- and interspecific comparisons and parameterization of numerical, physiological models exploring climate impacts [7,9]. Such standard protocols have been successfully applied for decades for other physiological traits, such as critical swimming speed [74,103] or hypoxia tolerance [75]. Additional research is needed on the *CT*_max_ of larvae and adults of more stenothermal (e.g. tropical or high latitude) species, which live much closer to their *CT*_*max*_, before developing any “universal *CT*_*max*_ protocol”. Moreover, the most suitable endpoint for these trials (e.g. loss of equilibrium, spasms, cessation of swimming) also needs to be carefully considered, especially for larvae. Finally, we wish to emphasize the importance of combining estimates of *CT*_*max*_ with measurements of thermal limits of other physiological processes (e.g. growth, metabolism) to account for acclimation potential and short- versus long-term thermal sensitivity to build a full picture of the thermal tolerance and performance curves of a species.

### Impact of acclimation temperature on *CT*_*max*_

Thermal acclimation is a type of phenotypic plasticity that occurs in many ectotherms [100]. This acclimation can be reversible (e.g. in response to diel or seasonal changes) or irreversible (in response to temperatures experienced during ontogeny). The plasticity of thermal tolerance due to acclimation has been examined by rearing at different, constant temperatures as well as at fluctuating (daily *in situ*) temperatures [104,105]. At either constant or fluctuating temperatures, and when experiencing increased temperature for either a short or long period of time (developmental temperature or heat pulses), exposure to warm temperatures subsequently increases tolerance to warmer temperatures (i.e. higher *CT*_*max*_). Hence, the increase in *CT*_max_ observed here for herring larvae reared (after the exogenous feeding stage) at 13°C compared to 7°C is not unexpected. Young herring larvae and eggs were reared at the same temperature to avoid any potential carry-over effects [106]. Therefore, one could suggest that the acclimation observed here is reversible (if larvae experienced prolonged, colder temperatures). However, this remains to be tested. In future studies, it will also be important to assess not only acclimation mechanisms [102] but also adaptive capacity since rapid increases in warming tolerance have been reported to occur within as little time as one generation in some species [107,108], although other reviews on ectotherms argue that plasticity in thermal tolerance is limited [109].

### Time dependency of thermal limits

Upper thermal limits of any species are largely time dependent [93,110]. One well-accepted concept to explain this time-limitation of thermal limits is the Oxygen- and Capacity-Limited Thermal Tolerance [110]. Beyond *T*_*pej*_, species start to experience the adverse effects of oxidative and thermal stress at the molecular level, which activates a suite of protective mechanisms (e.g. antioxidant and heat-shock responses, anaerobic metabolism). But these protective mechanisms are time-limited. Therefore, temperature tolerance is higher for shorter exposures and *vice versa*. A curvilinear relationship is expected between upper thermal limits and exposure time above *T*_*pej*_ [9], and this was observed in the data compiled on studies of the lethal limits (*LT50*_*max*_) of yolk sac larvae of Atlantic herring. The time-dependency was also evident for *CT*_*max*_ estimates of exogenously feeding herring larvae collected in the present study. It is unclear why *CT*_*max*_ estimates of yolk sac larvae in this study are similar to *LT50*_*max*_ values reported in other studies. Given the protocols and endpoints, one would expect the former to be higher than the latter. It could be that the temperatures of both endpoints are very similar or that it is simply difficult to make precise assessment of the loss of equilibrium in small larvae. Considering the present compilation, it is clear that much study is still needed on the time-limitation of thermal limits for different ontogenetic stages of fish if we wish to assess the impact of extreme events (e.g. heat waves) on populations.

## Conclusions

In the present study, we contribute to the growing body of literature on thermal limits of marine fish early life stages by examining the *CT*_*max*_ of Atlantic herring and European seabass and testing the effect of warming rate, a critical parameter of the dynamic method (i.e. ramping assay). In agreement with differences in the field distribution of these species, the larvae of herring had a lower *CT*_*max*_ (13.1–27.0– °C) than seabass (24.2–34.3°C). Importantly, warming rate had a species-specific impact on *CT*_*max*_, suggesting that future work on other species should first conduct pre-trials and then choose warming rates relevant for the context of the study. From a practical standpoint, the time dependency of survival at different, suboptimal cold or warm temperatures requires much additional study in order to understand the impact of extreme events (e.g. cold snaps, heat waves) on populations. The ultimate goal would be to compare this basic information on thermal limits (gained from a relatively rapid assay) with thermal performance curves for different traits (e.g. growth, swimming, feeding) which are more time consuming to obtain; clear relationships between longer-term thermal performance and short-term limits would improve confidence in making meaningful intra- (life stage-) and inter-specific comparisons of thermal sensitivity. It will be important to examine how each physiological trait is impacted by additional, interacting factors (e.g. *p*CO_2_, hypoxia). This information can be integrated within physiology-based models to make more robust (mechanistic) projections of how climate change will impact on the suitability of aquatic (marine and freshwater) habitats [111].

## Supporting information

S1 Fig Effect of body size on Critical Thermal Maxima (*CT*_*max*_) for Atlantic herring larvae. The y-axis represents the residuals from the Generalized Linear Model (GLM) presented in Fig 1. No significant effect of body size on *CT*_*max*_ was observed (see text). Symbols are shape-coded by acclimation temperature (circles, 7°C, triangles, 13°C).
(TIF)

S1 Table Significance of terms for the generalized linear model (GLM) on the impact of warming rate and acclimation temperature on Critical Thermal Maximum (*CT*_*max*_) in Atlantic herring larvae; and on the impact of body length on *CT*_*max*_ in European seabass larvae. (DOCX)
