# Supplementary material for: Effects of warming rate, acclimation temperature and ontogeny on the critical thermal maximum of temperate marine fish larvae
Source: PLoS One. 2017 Jul 27;12(7):e0179928. doi: 10.1371/journal.pone.0179928 (PMC5531428; doi:10.1371/journal.pone.0179928)
Supplement: S1 Table — (DOCX) [file pone.0179928.s002.docx]

**S1 Table. Significance of terms for the generalized linear model (GLM) on the impact of warming rate and acclimation temperature on Critical Thermal Maximum (*CT_max_*) in Atlantic herring larvae; and on the impact of body length on *CT_max_* in European seabass larvae.** Note that only feeding stages of both species are included in the models.

| **Atlantic herring** | | | |
| --- | --- | --- | --- |
|  | **DF** | **F-value** | **p-value** |
| Intercept | 1 | 34044.68 | <0.05 |
| Warming Rate | 4 | 18.09 | <0.05 |
| Acclim. Temperature | 1 | 15.78 | <0.05 |
| Warming rate : Temperature | 4 | 2.56 | <0.05 |
|  | | | |
| **European seabass** | | | |
|  | **DF** | **F-value** | **p-value** |
| Intercept | 1 | 24062.77 | <0.01 |
| Body Length | 1 | 59.51 | <0.01 |

Abbreviations: DF, degrees of freedom, Acclim., acclimation.
